# Could the mood disorder symptoms can be predict by metabolic disturbances?

**DOI:** 10.1192/j.eurpsy.2022.943

**Published:** 2022-09-01

**Authors:** J. Rog, M. Futyma-Jędrzejewska, D. Juchnowicz, R. Karpiński, H. Karakula-Juchnowicz

**Affiliations:** 1 Medical University of Lublin, 1st Department Of Psychiatry, Psychotherapy And Early Intervention, Lublin, Poland; 2 Prof. Mieczysław Kaczyński Neuropsychiatric Hospital in Lublin, Independent Public Healthcare Establishment, Lublin, Poland; 3 Medical University of Lublin, Department Of Psychiatric Nursing, Lublin, Poland

**Keywords:** Stress, lifestyle psychiatry, Depression, metabolic disturbances

## Abstract

**Introduction:**

Despite the huge progression in depression treatment, many individuals do not achieve full recovery. Studies demonstrated alternatives from neurotransmitter targets which are promising to predict and manage illness.

**Objectives:**

This study aimed to select metabolic factors linked to the severity of depression symptoms.

**Methods:**

66 patients (36% males) with episode of depression from part of SANGUT study were assessed for laboratory biomarkers (insulin, glucose, ALT, AST, lipid profile, cortisol, hs-CRP), anthropometric measurements (BMI, body composition, WHR ratio) and severity of subjective depressive (BDI scale) and stress (PSS-10 scale) symptoms.

**Results:**

Maximum accuracy for differentiating mood symptoms was achieved by the combination of triglycerides (cut-off point > 101 mg/dl) and HDL cholesterol (cut-off point <=48 mg/dl). For differentiating stress symptoms the combination of cholesterol LDL (cut-off point > 108.35 mg/dl) and hs-CRP (cut-off point <=1.55 mg/dl) were most accurate. In the regression analysis model, total; LDL and HDL cholesterol, adjusting for HOMA-ir, cortisol, hs-CRP, triglycerides, age and body fat content were independently related to mood symptoms severity and explain 23.4% variability. Stress symptoms were related to cortisol, hs-CRP levels and WHR ratio adjusted for age, duration of illness, LDL cholesterol, and body fat content. The following model explains 19% variability of symptoms severity.

**Conclusions:**

In patients with mood disorders, more attention should be paid to metabolic changes, predicting intensified depression traits. The results indicate lifestyle changes as an available to all patients tool for depression management.

**Disclosure:**

No significant relationships.

