# Could the mood disorder symptoms can be predict by metabolic disturbances? – CORRIGENDUM

**DOI:** 10.1192/j.eurpsy.2025.17

**Published:** 2025-02-21

**Authors:** J. Rog, M. Futyma-Jędrzejewska, D. Juchnowicz, R. Karpiński, H. Karakula-Juchnowicz

This abstract was originally published with a missing affiliation for author R. Karpiński. He is affiliated with both Chair and I Department of Psychiatry, Psychotherapy and Early Intervention, Medical University of Lublin, 20-059 Lublin, Poland, and Department of Machine Design and Mechatronics, Faculty of Mechanical Engineering, Lublin University of Technology, Lublin, Poland.

This has now been corrected in the abstract and this corrigendum published.

## References

[1] Rog J, Futyma-Jędrzejewska M, Juchnowicz D, Karpiński R, Karakula-Juchnowicz H. Could the mood disorder symptoms can be predict by metabolic disturbances? European Psychiatry. 2022; 65(S1):S371–372.10.1192/j.eurpsy.2025.1739980077

